# Efficacy and Safety of Chuanxiong Qingnao Granule in Patients with Migraine: A Randomized, Double-Blind, Placebo-Controlled Trial

**DOI:** 10.1155/2021/6203999

**Published:** 2021-12-22

**Authors:** Yi-Fan Li, Hui-Min Hu, Bo-Ning Wang, Yi Zhang, Xing Liu, Peng Mao, Bi-Fa Fan

**Affiliations:** ^1^Graduate School of Beijing University of Chinese Medicine, Beijing 100029, China; ^2^Department of Pain Medicine, China-Japan Friendship Hospital, Beijing 100029, China

## Abstract

**Objective:**

To evaluate the efficacy and safety of Chuanxiong Qingnao Granule (CQG) to treat migraine.

**Method:**

This study was a randomized, double-blind, placebo-controlled trial. All migraineurs were recruited and randomly assigned into a treatment group treated with CQG and a control group treated with a placebo. The whole research process included a 4-week baseline, 12-week intervention, and 12-week follow-up. The primary outcome was responder rate, defined as the percentage of migraineurs with 50% or more reduction in the frequency of migraine attack during treatment and posttreatment period compared with the baseline. The secondary outcomes were the number of migraine days, migraine attack frequency, visual analogue scale (VAS), Fatigue Severity Scale (FSS), Hamilton Depression Scale (HAMD), and Migraine Disability Assessment (MIDAS).

**Results:**

A total of 346 migraineurs completed the research and were included in the intention-treatment analyses. The response rates differed significantly between the treatment group and the control group (71.5% vs. 12.1% at week 12 and 83.1% vs. 3.4% at week 24). Attack frequency, days of headache attack, VAS, FSS, HAMD, and MIDAS decreased at week 12 in both groups with more reduction in the treatment group (*P* < 0.001). No severe adverse events were observed in this trial.

**Conclusion:**

Chuanxiong Qingnao Granule can significantly improve headache symptoms in patients with migraine while improving disability, fatigue, and depression with a good safety profile.

## 1. Introduction

Migraine, a prevalent neurological disorder, is characterized by recurrent episodes of moderate-to-severe pulsating headache associated with neurological, gastrointestinal, and sensory dysfunction [1]. In China, the prevalence of migraine is about 0.9% [2], which is considerably lower than reality because of a low level of disease awareness [3]. Migraine is a highly burdensome condition for patients, families, and society, which is induced by both direct cost (consumption of health care resources) and indirect cost (loss of productivity) of migraine [4].

However, the underlying pathogenesis of migraine is not clearly understood, which hinders the development of mechanism-based therapies. The pharmacologic approach to managing migraine is dichotomously categorized into abortive treatment and preventive treatment [5]. First-line acute therapy options mainly include nonsteroidal anti-inflammatory drugs (NSAIDs) and triptans, of which only the latter are specific for the condition [6]. However, comorbidities like coronary artery disease, peripheral arterial disease, and stroke preclude specific rescue drugs, especially for older people who are more susceptible to adverse effects of medication [7, 8]. Preventive medicines mainly include *β*-blockers, antidepressants, and antiepileptic drugs [9].

Recently, increased interest has been devoted to the study of calcitonin gene-related peptide (CGRP), a neuromodulator and vasodilator peptide that plays a vital role in the occurrence of migraine. Several monoclonal antibodies which bind to CGRP receptor or ligand have been approved by US Food and Drug Administration (FDA) but have not been widely promoted as first-line treatment in various countries and regions [10, 11].

In addition, interventional therapy also has a good effect on migraine. Due to the lack of clear understanding of the nerves involved, the primary surgical intervention is stellate ganglion block (SGB). As an invasive treatment, SGB increases the risk of complications such as bleeding, infection, recurrent laryngeal nerve injury, and pneumothorax, which can cause panic and rejection in patients [12]. In addition, in the context of the COVID-19 epidemic, the hospitalization rate and invasive treatment should be minimized to reduce the risk of nosocomial infection.

As a result, there has been a dire need for novel therapies that provide sustained efficacy and good tolerability to enable long-term adherence and improve the impact of migraine on patients' life. Traditional Chinese medicine (TCM), rooted in natural ingredients, is more acceptable for migraineurs while meeting the need for controlling pain and associated bothersome symptoms of migraine [13]. Chuanxiong Qingnao Granule is a Chinese patent medicine for both the prevention and the treatment of migraine and has been widely used in clinical practice in China. However, evidence-based multicenter clinical research on the efficacy and safety of the CQG is still lacking. This study adopted a randomized, double-blind, placebo-controlled experimental design to test the efficacy and safety of Chuanxiong Qingnao Granules in treating migraine.

## 2. Methods

### 2.1. Study Design and Oversight

#### 2.1.1. Sample Size

A randomized, double-blind, placebo-controlled clinical trial was conducted with migraine patients enrolled from China-Japan Friendship Hospital in Beijing of China from January 2017 to December 2019. The diagnosis of migraine in patients was made by a pain physician. Patients who met the diagnosis were enrolled in the study and arranged to complete blood routine, biochemical examinations, and electrocardiogram. If laboratory test results met the exclusion criteria, we would contact the patient to withdraw from the study. According to the preexperiment with a small sample, with 50% or more reduction in headache frequency as the primary outcomes, it was estimated that responder rate in the treatment group was 80% and that in the control group was 50%. With *α* = 0.05 (bilateral) and power = 0.90, PASS11 software suggested the treatment group *N*1 = 48 and the placebo group *N*2 = 48. Assuming that the loss to follow-up rate was 20%, the adjusted sample sizes were NI = 60 and *N*2 = 60, respectively. In the present study, 400 migraineurs were enrolled before the 4-week baseline period.

#### 2.1.2. Random Methods

There was stratified randomization by the hospital. The random codes were generated by SAS software using stratified block randomization to ensure that the numbers of participants between the two groups were almost equal (1 : 1).

#### 2.1.3. Blinding

A double-blind design was adopted, and CQGs and placebos were repackaged and distributed by the standardized operating steps of a double-blind clinical trial. After the blind bottom was sealed, it was handed over to the clinical research agency for proper storage. Blind review, first-level unblinding, and statistical analysis were carried out after case collection.

The whole research process included a 4-week baseline, 12-week intervention, and 12-week follow-up. The study protocol complied with the World Medical Association's Declaration of Helsinki and China's regulations and guidelines for good clinical practice. It was approved by the ethics committee of the hospital. All participants provided written informed consent.

### 2.2. Inclusion Criteria

Participants who met all the following requirements were allowed for enrollment: (1) migraine with a typical aura or without aura, either of which fulfilled the International Classification of Headache Disorders 2nd edition for migraine strictly [14]; (2) visual analogue scale (VAS) ≥4; (3) migraine attack frequency during the baseline period being at least 2 times per month; (4) aged 18–60 years; (5) stopped other migraine treatment drugs for more than 2 weeks before enrollment; (6) provided written informed consent.

### 2.3. Exclusion Criteria

Participants who met one of the following were excluded: (1) special types of migraine, such as ophthalmoplegia migraine, hemiplegic migraine, and migraine with brainstem aura; (2) 10 days or more analgesic taking monthly; (3) severe comorbidities such as cardiovascular system disease, cerebrovascular disease, intracranial space-occupying lesions, and/or gastrointestinal, renal, hepatic, hematologic, respiratory, or endocrine disease, or psychosis; (4) abuse of alcohol or other drugs; (5) allergy to CQG; (6) pregnancy and lactation.

### 2.4. Interventions

Participants in the experimental group were given CQGs, while the control group was given simulant placebos, which looked in shape and color similar to CQG. Both placebo and CQG contained excipients, which were composed of plant volatile oils, essences, and other ingredients to improve the taste of the medicine, mask the smell of herbal products, and make the taste and smell of the two as similar as possible. Both CQG and placebo were made by *Jichuan Pharmaceutical Group Co., Ltd*. CQG was composed of 14 kinds of herbs, including *Rhizoma Chuanxiong* (Chuanxiong), *Radix Angelicae Sinensis* (Danggui), *Radix Saposhnikoviae* (Fangfeng), *Radix Angelicae Dahuricae* (Baizhi), *Radix Ophiopogonis* (Maidong), *Notopterygium* (Qianghuo), *Radix Angelicae Pubescentis* (Duhuo), *Rhizoma Atractylodis* (Cangzhou), *Flos Chrysanthemi* (Juhua), *Fructus Viticis* (Manjingzi), *Radix Scutellariae* (Huangqin), *Radix et Rhizoma Glycyrrhizae* (Gancao), and *Rhizome Zingiberis* (Ganjiang). All CQGs and placebos were packed in a small bag which contained a dose of 10 g. Each subject took 10 g of CQG or placebo three times a day for 12 weeks. As for intolerable headaches, the patients were instructed to take Ibuprofen (300 mg capsules with sustained release) as a rescue medication, and the usage of it was recorded in the headache diary.

### 2.5. Outcome Measures

All patients were required to keep a headache diary every day. The physicians evaluated and recorded the characteristics of migraine and adverse events during the baseline period, treatment period, and posttreatment period. Evaluation of therapeutic effects was performed at the end of the follow-up.

The primary outcome measure was responder rate, defined as the percentage of patients with 50% or more reduction in the frequency of migraine attack during treatment and posttreatment period compared with the baseline period.

The secondary outcome measures were migraine days, migraine attack frequency, visual analogue scale (VAS), Fatigue Severity Scale (FSS), Hamilton Depression Scale (HAMD), and Migraine Disability Assessment (MIDAS). The VAS is a 100 mm continuous horizontal line with the 0 mm end meaning no pain and the 100 mm end representing the most severe pain; the in-between part means different degrees of pain [15]. FSS is a scale for evaluating the fatigue level of patients, which consists of 9 items on a scale of 1 to 7, with 1 indicating “strongly disagree” and 7 indicating “strongly agree” [16]. HAMD is a standard depression scale that assesses the severity of depression in patients from various aspects such as somatization symptoms, sleep disturbance, cognitive impairment, and weight loss. The scale used in this study contained a total of 24 items, each of which had 0 points for asymptomatic, 1 point for mild, 2 points for moderate, 3 points for severe, and 4 points for very severe. The higher the total score, the greater the degree of depression [17]. MIDAS questionnaire is used to evaluate the extent to which migraine interferes with the capability to carry out responsibilities and functions in daily life. It is composed of five questions that are scored to convert to a MIDAS disability grade and two other questions that concentrate on the frequency and severity of migraine [18]. The MIDAS score is the sum of the number of days absent from work and the number of days when work efficiency is reduced by half or more. The score is divided into 4 levels: 0∼5 indicating little or no disability; 6∼10 indicating mild disability; 11∼20 indicating moderate disability; 21 or higher indicating severe disability.

In the present trial, safety and tolerability assessments included vital signs, ECG, clinical laboratory tests (blood routine and blood chemical values), and adverse events. The date of onset, resolution date, severity, frequency, relationship to study treatment, action taken, and outcome of adverse events were recorded in detail.

### 2.6. Statistical Analyses

Statistical analysis was performed using the IBM SPSS 19.0. Descriptive statistical results were shown as mean ± standard deviation for continuous data, number, and percentage for categorical data. In comparison between groups, chi-square tests were used for categorical data, and *t*-tests or rank-sum tests were used for continuous data depending on whether the data met the normal distribution and homogeneity of variance. A nonparametric ANOVA (Kruskal–Wallis H test) followed by multiple pairwise comparisons was used to compare the pre-/post- and pretreatment/follow-up differences of the same group. *P* < 0.05 indicates that the difference is statistically significant.

## 3. Results

### 3.1. Patients

**Figure 1:**
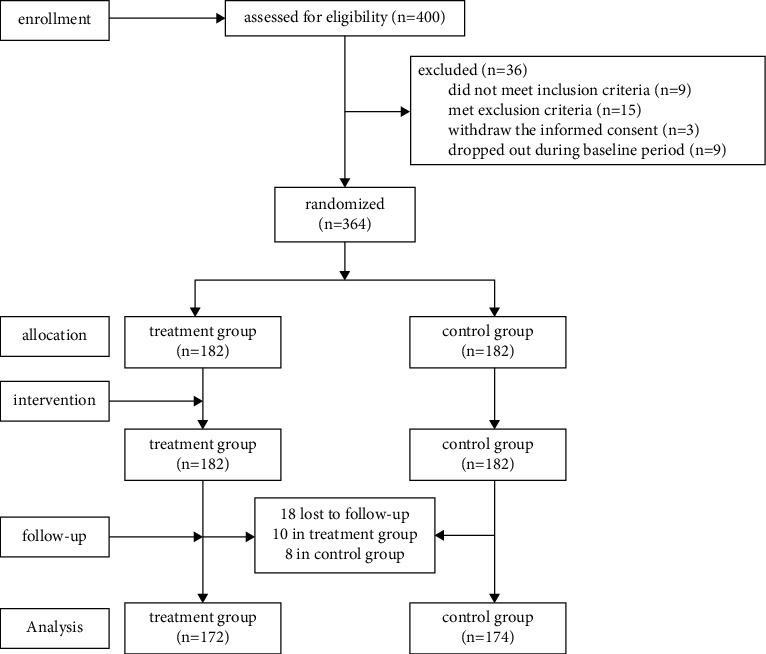
Experimental flowchart.

400 eligible Chinese patients with migraine were screened, and 36 patients were excluded during baseline. Among 36 cases, 9 met migraine attack frequency during the baseline period less than 2 times per month, 3 withdrew the informed consent, 9 could not finish the pain diary during baseline, 1 was pregnant at baseline, and 14 were found to have serious comorbidities. The remaining 364 were randomly assigned to either treatment group (*n* = 182) or control group (*n* = 182). In the course of this trial, 18 cases were dropped (10 cases in the treatment group, 8 cases in the control group), and a total of 346 patients completed the study. It was included in the intention-to-treatment analyses (172 cases in the treatment group, 174 cases in the control group) (see Figure 1). Participants had a mean age of 41.92 years, and most were females (65.9%; *n* = 228). Baseline demographics of sex, age, marital status, education, monthly income, and past illnesses had no statistical difference in either group (*P* > 0.05). Patients were diagnosed as having migraine for 16.57 years before study enrollment on average, and most of them were diagnosed with migraine without aura (79.8%; *n* = 276) and unilateral headache (78.9%, *n* = 273) (see Table 1). As for efficacy measures of baseline participants, there were no significant differences in frequency of headache attack, days of headache attack, VAS, Fatigue Severity Scale, Hamilton Depression Scale, or Migraine Disability Assessment (*P* > 0.05) (see Table 2).

### 3.2. Primary Outcomes

The responder rates in treatment vs. control groups were 71.5% vs. 12.1% after 12 weeks (*P* < 0.001) and 83.1% vs. 3.4% at follow-up period (*P* < 0.001). The treatment group was significantly superior to the control group in responder rate (see Tables 2 and 3). Table 1 shows the uneven distribution of gender and duration between the two groups, which have a confounding role in migraine. Therefore, considering group, duration, and gender as independent variables, and primary outcome as a dependent variable, binary logistic regression analysis was performed to correct confounding factors. The corrected results suggest that OR <1 and the results of this study are meaningful (OR = 0.006, 95%CI: 0.002–0.016). See Supplementary Materials for details (available here).

### 3.3. Secondary Outcomes

#### 3.3.1. Frequency of Headache Attack

**Figure 2:**
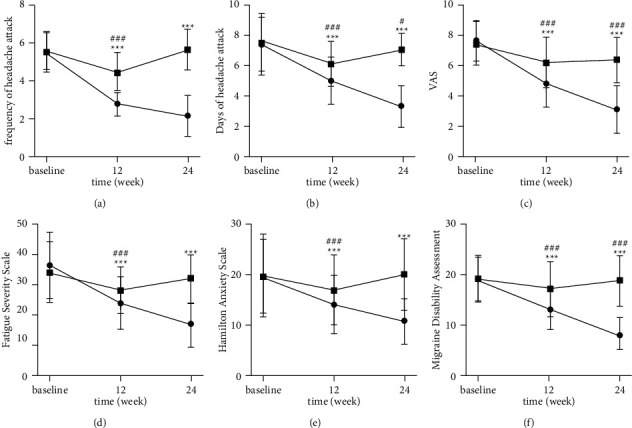
^*∗∗∗*^ *P* < 0.001 for treatment vs. baseline; ^###^*P* < 0.001 for control vs. baseline; ^#^*P* < 0.05 for control vs. baseline; absence of label indicates a nonsignificant difference from the baseline (*P* > 0.05). CG: control group; TG: treatment group ● represents treatment group (TG); ◾ represents control group (CG).

Headache frequency in treatment group and control group decreased to 2.81 ± 0.69 and 4.45 ± 1.03 at week 12, respectively. And, compared with the baseline period, the differences are statistically significant (*P* < 0.001; see Table 2 and Figure 2). After the drug was discontinued, the frequency of headaches in the treatment group was further reduced from 2.81 ± 0.69 to 2.16 ± 1.09. In contrast, that of the control group returned to a level similar to the baseline period (see Table 2 and Figure 2).

#### 3.3.2. Days of a Headache Attack

During the study period, migraine days were descending from 7.35 to 3.31 gradually in the treatment group. The difference in days of headache attack between the two groups was significant at the treatment period (*P* < 0.001) and follow-up period (*P* < 0.001) (see Tables 2 and 3). Days of headache attacks in the control group at week 24 were more significant than those at week 12 but were less than those at the baseline period (see Figure 2).

#### 3.3.3. VAS

The VAS scores decreased significantly at week 12 and week 24 in both groups when compared with baseline (*P* < 0.001) while the group gaps grew (see Figure 2(c)). The VAS scores in the treatment group were significantly lower compared with the control group at week 12 and week 24, respectively (4.81 ± 1.57 vs. 6.17 ± 1.65, *P* < 0.001; 3.12 ± 1.57 vs. 6.37 ± 1.48, *P* < 0.001) (see Tables 3 and 4).

#### 3.3.4. FSS

The differences in FSS between the treatment group and the placebo group were significant at both time points (23.34 ± 8.36 vs. 27.29 ± 7.62, *P* < 0.001; 15.98 ± 7.18 vs. 30.77 ± 7.92, *P* < 0.001) (see Tables 3 and 4). At week 24, the treatment group still had a downward trend while the control group returned to the baseline level (see Figure 2(d)).

#### 3.3.5. HAMD

Decreased HAMD scores were observed in the treatment group when compared with the control group (14.13 ± 5.55 vs. 16.78 ± 6.79, *P* < 0.001; 10.85 ± 4.46 vs. 19.13 ± 4.97, *P* < 0.001) (see Tables 3 and 4). HAMD scores in the treatment group at weeks 12 and 24 were significantly decreased compared with the baseline (14.13 ± 5.55 vs. 19.62 ± 7.95, *P* < 0.001; 10.85 ± 4.46 vs. 19.62 ± 7.95, *P* < 0.001) (see Figure 2(e)).

#### 3.3.6. MIDAS

There were significant differences between the treatment group and the control group at weeks 12 and 24 (*P* < 0.001). The MIDAS scores in the treatment group decreased more than those in the control group and continued to decline during the follow-up period (see Figure 2(f)).

#### 3.3.7. Usage of Acute Analgesics

The number of patients using acute analgesics in the two groups at baseline was similar, while it significantly differed both at treatment and at follow-up. Treatment group had reduced acute medicine compared with control group after intervention (see Table 5).

### 3.4. Safety Assessments

During a 12-week intervention, 7 patients reported mild nausea symptoms after taking medicine, including 5 in the treatment group and 2 in the control group. After causes such as gastrointestinal system damage were excluded, the patients were advised to take the drug after meals, and then the symptom was relieved or disappeared. There were no significant abnormal changes in blood routine, blood chemical value, or electrocardiogram in two groups, and no serious adverse events occurred.

## 4. Discussion

This study provides evidence for the benefit of CQG in the prevention and treatment of migraine. Outcome measures illustrated that CQG greatly reduced the frequency, days, and degree of headache compared with placebo. Notably, the data during the follow-up period indicated that CQG still had a relatively stable therapeutic effect after drug withdrawal. During the intervention period, all patients' demand for acute analgesics gradually decreased. We found that most patients had rarely used analgesics after taking CQG for 12 weeks. It means that CQG may reduce the occurrence of medication overuse headaches. According to our findings, CQG should be considered as an option for the prevention and treatment of migraine. Alternatively, CQG, as a complementary therapy to combine with another medicine or surgery, is also beneficial.

A review mentioned that migraine might be linked with fatigue and mood disorders by specific shared biological mechanisms via brain regions and neurotransmitter pathways [19]. This study observed that CQG not only improved headache symptoms but also had a good long-term effect on patients' fatigue and mood disorders. In addition, CQG could help patients retrieve normal daily work, life, and social activities, which not only reduced the consumption of healthcare but also restored personal productivity. We did not independently observe the subjects' sleep condition, but HAMD contained a brief assessment of sleep. We found that most patients got better sleep after taking CQG. Therefore, CQG may be used as a complementary treatment to improve these accompanying symptoms.

A growing body of evidence supports that CQG and its herbal ingredients can improve the frequency of migraine attacks and the severity of pain. Literature research revealed that CQG combined with Flunarizine could significantly decrease 5-HT in platelets and increase *β*-endorphin in blood while slowing down the blood flow speed of the middle cerebral artery (MCA) and anterior cerebral artery (ACA) [20]. According to modern pharmacological research, CQG can also reduce blood viscosity, inhibit vasoconstriction, and, at the same time, improve blood circulation in the brain and the state of ischemia and hypoxia and protect brain tissue cells from oxidative damage [21]. The volatile oil components of *Angelicae Dahuricae* can increase the expression of brainstem melanin mRNA and improve the content of endogenous analgesic substances, thereby activating the endogenous analgesic mechanism and exerting its analgesic effect [22]. In a GTN-induced migraine model, it was reported that Baizhi and chuanxiong could alleviate migraine by regulating abnormal levels of the proinflammatory factor, neurotransmitters, and vasoactive substances [23].

Before this study, there were many similar types of research on the treatment and prevention of migraine in TCM, with pros and cons. A multicenter double-blind, randomized, placebo-controlled clinical trial demonstrated that Tianshu capsule (TSC), a formula of traditional Chinese medicine, could decrease the frequency and days of migraine and improve related symptoms with good safety [24]. In this trial, primary and second outcomes included only the directly related indicators of headache, such as headache frequency, VAS, and number of onset days, while evaluation of mood, sleep status, quality of life, and disability status was lacking. However, migraine is a complex nervous system function affecting people physically and mentally rather than simply a vascular headache. A randomized clinical trial of acupuncture intervention for migraine reported that true acupuncture manifested persisting superiority and clinically relevant benefits for 24 weeks in migraine prophylaxis compared with sham acupuncture [25]. Patients undergoing acupuncture need to go to the hospital repeatedly for time-consuming treatment, and some patients may drop out of the treatment due to fear of needles or fainting.

In the safety analysis, 7 patients in this study reported mild nausea symptoms after taking CQG, and the symptoms were relieved after the medication time was adjusted. We consider this related to the components of various herbal volatile oil contained in this medicine and individual taste preferences, which may guide the optimization of the extraction process of the active ingredients of the medicine and dosage form.

Several strengths should be mentioned in the present study. To the best of our knowledge, this is the first TCM study for migraine that incorporates Fatigue Severity Scale (FSS) and Hamilton Depression Scale (HAMD) into outcomes. The risk of depression in migraine patients is 2 to 4 times that of nonmigraine patients, and the risk of migraine in patients with depression is also about 2 to 4 times that of nondepression patients [26]. In a Korean hospital-based study, nearly half of patients indicated that fatigue is the cause of headaches [27]. Given that migraine, fatigue, and depression are probably two-way related diseases rather than simple comorbidities, treating fatigue and mood complaints is likely to form an essential aspect of migraine management.

Short follow-up time, no animal experiment support, and unknown mechanisms of individual herbs in CQG are the shortcomings of this trial. Considering the patient's compliance, the follow-up period did not exceed 12 weeks, which prevented the tracking of the long-term efficacy of CQG and the recurrence. Practical experiments should be designed to clarify the mechanism and side effects of each herb component of CQG in the future.

## 5. Conclusion

Chuanxiong Qingnao Granule can significantly alleviate headache symptoms in patients with migraine while improving disability, fatigue, and depression with a good safety profile.

## Figures and Tables

**Table 1:** General information of patients in both groups.

Characteristics	Treatment (*n* = 172)	Control (*n* = 174)	*P* value
*Gender, n (%)*			
Male	50 (29.1)	68 (39.1)	
Female	122 (70.9)	106 (60.9)	0.050
Age, mean (SD), years	41.36 (9.16)	42.47 (7.95)	0.325

*Marital status, n (%)*			
Unmarried	27 (15.7)	40 (23.0)	
Married	145 (84.3)	134 (77.0)	0.086

*Education, n (%)*			
Bachelor degree and above	27 (15.7)	29 (16.7)	
Senior high school	47 (27.3)	53 (30.4)	
Junior high school	40 (23.3)	37 (21.3)	
Primary school or below	58 (33.7)	55 (31.6)	0.893

*Monthly income, n (%)*			
<500 RMB	3 (1.7)	8 (4.6)	
500–1000 RMB	34 (19.8)	26 (14.9)	
1001–3000 RMB	57 (33.2)	58 (33.4)	
3001–5000 RMB	58 (33.7)	60 (34.5)	
>5000 RMB	20 (11.6)	22 (12.6)	0.483

Smoking, *n* (%)	67 (39.0)	76 (43.7)	0.372
Alcohol, *n* (%)	56 (32.6)	50 (28.7)	0.441
Stroke, *n* (%)	9 (5.2)	17 (9.8)	0.109
Hypertension, *n* (%)	86 (50.0)	91 (52.3)	0.669
Diabetes, *n* (%)	57 (33.1)	66 (37.9)	0.352
Hyperlipemia, *n* (%)	66 (38.4)	78 (44.8)	0.223
Heart disease, *n* (%)	35 (20.3)	47 (27.0)	0.145
*Diagnosis, n (%)*			
Migraine with aura	28 (16.3)	42 (24.1)	
Migraine without aura	144 (83.7)	132 (75.9)	0.069
Duration of migraine (year)	15.60 ± 8.94	17.52 ± 8.74	0.040

*Location of headache, n* (%)			
Left	74 (43.0)	72 (41.4)	
Right	64 (37.2)	63 (36.2)	
Bilateral	34 (19.8)	39 (22.4)	0.833

**Table 2:** Headache characteristics of participants in the baseline period.

Characteristic	Treatment (*n* = 172)	Control (*n* = 174)	*P* value
Frequency of headache attack, mean (SD)	5.53 ± 1.01	5.55 ± 1.09	0.813
Days of headache attack, mean (SD)	7.35 ± 1.90	7.57 ± 1.87	0.236
VAS, mean (SD)	7.64 ± 1.38	7.48 ± 1.43	0.264
FSS, mean (SD)	35.00 ± 10.49	32.93 ± 9.85	0.132
HAMD, mean (SD)	19.62 ± 7.95	19.52 ± 7.08	0.767
MIDAS, mean (SD)	19.46 ± 4.42	19.63 ± 4.67	0.722

VAS: visual analogue scale; FSS: Fatigue Severity Scale; HAMD: Hamilton Depression Scale; MIDAS: Migraine Disability Assessment.

**Table 3:** Efficacy measurements of participants at week 12.

Characteristic	Treatment (*n* = 172)	Control (*n* = 174)	*P* value
Percentage of patients with ≥50% reduction in frequency of headache, *n* (%)	123 (71.5)	21 (12.1)	<0.001
Frequency of headache attack, mean (SD)	2.81 ± 0.69	4.45 ± 1.03	<0.001
Days of headache attack, mean (SD)	4.99 ± 1.54	6.09 ± 1.48	<0.001
VAS, mean (SD)	4.81 ± 1.57	6.17 ± 1.65	<0.001
FSS, mean (SD)	23.34 ± 8.36	27.29 ± 7.62	<0.001
HAMD, mean (SD)	14.13 ± 5.55	16.78 ± 6.79	<0.001
MIDAS, mean (SD)	13.73 ± 4.29	17.52 ± 5.51	<0.001

**Table 4:** Efficacy measurements of participants at week 24.

Characteristic	Treatment (*n* = 172)	Control (*n* = 174)	*P* value
Percentage of patients with ≥50% reduction in frequency of headache, *n* (%)	143 (83.1)	6 (3.4)	<0.001
Frequency of headache attack, mean (SD)	2.16 ± 1.09	5.68 ± 1.10	<0.001
Days of headache attack, mean (SD)	3.31 ± 1.40	7.05 ± 1.05	<0.001
VAS, mean (SD)	3.12 ± 1.57	6.37 ± 1.48	<0.001
FSS, mean (SD)	15.98 ± 7.18	30.77 ± 7.92	<0.001
HAMD, mean (SD)	10.85 ± 4.46	19.84 ± 6.89	<0.001
MIDAS, mean (SD)	8.40 ± 3.32	19.13 ± 4.97	<0.001

**Table 5:** Usage of acute analgesics, *n* (%).

	Treatment	Control	*P* value
Baseline	78 (45.3)	83 (47.7)	0.661
1–4 weeks	25 (14.5)	48 (27.6)	0.003
5–8 weeks	18 (10.5)	43 (24.7)	0.001
9–12 weeks	26 (15.1)	57 (32.8)	0.000
13–16 weeks	23 (13.4)	69 (39.7)	0.000
17–20 weeks	31 (18.0)	61 (35.1)	0.000
21–24 weeks	27 (15.7)	58 (33.3)	0.000

## Data Availability

The data used to support the findings of this study are restricted by the ethics committee of China-Japan Friendship Hospital in order to protect patient privacy. Data are available only for researchers who meet the criteria for access to confidential data.

## Supplementary Materials

Supplementary Materials Table 6: binary logistic regression analysis results about confounding factors.
