# Supplementary material for: Efficacy and Safety of Chuanxiong Qingnao Granule in Patients with Migraine: A Randomized, Double-Blind, Placebo-Controlled Trial
Source: Evid Based Complement Alternat Med. 2021 Dec 22;2021:6203999. doi: 10.1155/2021/6203999 (PMC8716194; doi:10.1155/2021/6203999)
Supplement: Supplementary Materials — Table 6: binary logistic regression analysis results about confounding factors. [file 6203999.f1.docx]

Table 6 Binary Logistic Regression Analysis Results about Confounding Factors

|  | OR（95%CI） | P-value |
| --- | --- | --- |
| Group  Treatment group  Control group | 1.00  0.006(0.002-0.016) | 0.000 |
| Gender  Male  Female | 1.00  1.247(0.574-2.710) | 0.577 |
| Duration | 1(0.960-1.042) | 0.998 |
